# Supplementary material for: Assessing Africa’s child survival gains and prospects for attaining SDG target on child mortality
Source: PLOS Glob Public Health. 2024 Jul 10;4(7):e0003022. doi: 10.1371/journal.pgph.0003022 (PMC11236144; doi:10.1371/journal.pgph.0003022)
Supplement: Attachment — Submitted filename: Response to Reviewers_PLOS_GPH.docx [file pgph.0003022.s001.docx]

**Response to Reviewers’ Comments**

PGPH-D-23-02289
Assessing Africa’s child survival gains and prospects for attaining SDG target on child mortality
PLOS Global Public Health

**Comments**
Thank you for submitting your manuscript to PLOS Global Public Health. After careful consideration, we feel that it has merit but does not fully meet PLOS Global Public Health’s publication criteria as it currently stands. Therefore, we invite you to submit a revised version of the manuscript that addresses the points raised during the review process.

**Response**

Thank you

**Comments**
It is an interesting study where authors investigated Africa’s child survival gains and prospects for attaining SDG target on child mortality. However, some technical issues should be addressed further, especially English language as mentioned by the reviewers.

**Response**

Thank you for the comments. We have done a thorough editing of the entire manuscript and improved the English expression.

Reviewers' comments:
Reviewer #1:

**Comments**

I have read the referred article with keen interest. The information is interesting and innovative; conclusion section is interesting and authors can improve it further. I am recommending authors to do a little more work and add latest literate to support the study. The authors need to improve results section. The level of English is good and smooth, e.g., the language standard, specifically the grammar, of sufficient quality to meet scientific merit for publication. However, I suggest authors to double check for language quality.

**Response**

As pointed out, thorough editorial work has been done on all sections pf the manuscript to improve the language quality.

**Comments**
Describe scientific contribution of the study to the existing body of knowledge.

**Response**

Thank you for the suggestion. We have further described the scientific contribution of our study to advance the existing knowledge.

**Comments**
I endorse this manuscript after minor revision as suggested. The topic is interesting and worthy of attention. The methodology is adequate and the conclusions are consistent with the reported data. The manuscript can be improved by expanding the references and citing some recently published articles on this topic.

**Response**

Thank you for the comments. Additional relevant references have been reviewed and included in the introduction and discussion sections of the reviewed manuscript.

Reviewer #2:

**Comments**
The paper cannot be considered for publication unless it is rewritten in proper English. Methodology and results sections sound good but there are so many linguistic errors that makes full understanding the paper not possible.

**Response**

Thank you for the comments. To improve the English expression, we have carried out thorough editing of the manuscript.
